# AN EXPLICIT LIVE DISCHARGE PROTOCOL (LDP) TO IMPROVE CARE TRANSITIONS

**DOI:** 10.1093/geroni/igae098.1797

**Published:** 2024-12-31

**Authors:** Stephanie Wladkowski, Susan Enguidanos, Tracy Schroepfer

**Affiliations:** Bowling Green State University, College of Health & Human Services, Bowling Green, Ohio, United States; University of Southern California, Los Angeles, California, United States; University of Wisconsin Madison, Madison, Wisconsin, United States

## Abstract

Discharge planning for care transitions is required in hospitals, skilled nursing facilities, and home health agencies within the United States under Medicare guidelines. Yet, no required discharge planning or clear guidelines are available to guide practitioners in transitioning patients and their caregivers out of hospice care. A live discharge from hospice can occur when a patient stabilized under hospice care no longer meets the life expectancy hospice eligibility criteria. For these individuals and their caregivers, the result is a disruption of care continuity. Further, an increased burden is placed on primary caregivers who may be unprepared for this transition. Presenters have developed a live discharge protocol (LDP), to standardize the live discharge process and assess for psychosocial support needs for patients and their caregivers post-discharge. This presentation will describe the development of the LDP through several steps: first, with an advisory committee made up of research experts, clinicians, and caregivers. Next, through focus groups to gain a deeper understanding of current approaches and accompanying challenges of a discharge protocol. Finally, the LDP was drafted and reviewed by practicing social workers and nurses for usability. Further, this presentation will report on how each step provided valuable feedback and informed the final LDP currently being used by hospice agencies and the complexities of conducting a live discharge, the uniqueness of each hospice agency, and the need for more research to support a standardized and reimbursable discharge process.

